# Fostering a Trusting Relationship With Family in Dementia Special Care Units: A Participatory Action Research Project

**DOI:** 10.1111/jan.16432

**Published:** 2024-09-04

**Authors:** Nina Hovenga, Elleke Landeweer, Ivonne Lesman‐Leegte, Sacha Van Twillert, Floor Vinckers, Sytse Zuidema, Carlo Leget

**Affiliations:** ^1^ Department of Primary‐ and Long‐Term Care University of Groningen, Faculty of Medical Sciences, University Medical Center Groningen Groningen The Netherlands; ^2^ Department of Care Ethics University of Humanistic Studies Utrecht The Netherlands; ^3^ UMC Staff Policy and Management Support University of Groningen, University Medical Center Groningen Groningen The Netherlands

**Keywords:** dementia special care unit, family involvement, narrative analysis, nurses, nursing home staff, participatory action research, trust

## Abstract

**Aim:**

To explore experiences of nursing home staff in implementing self‐designed interventions to foster trusting relationships with family in practice.

**Design:**

This qualitative study used a Participatory Action Research approach.

**Methods:**

Data collection included focus groups (*n* = 15), interviews (*n* = 28) and observations (*n* = 5). A holistic narrative approach was used for data analysis, resulting in co‐constructed narratives representing experiences of nursing home staff in implementing four different interventions in five Dutch dementia special care units in nursing homes. The data collection period began in August 2021 and ended in April 2022.

**Results:**

Nursing home staff implemented self‐designed interventions to foster trusting relationships with family, including initiating informal conversations, sharing residents’ ‘happy’ moments, discussing mutual expectations, and being more aware of families’ emotional burdens. Identified facilitators emphasise the importance of reciprocity, familiarity, transparency, realistic goal setting and empathy. Identified barriers are related to moral uncertainty in balancing competing demands, conflicting social norms, prioritising hands‐on care tasks over family contact and lack of courage to act.

**Conclusion:**

Nursing home staff conclude that their interventions contribute positively to building and maintaining a trusting relationship with families.

**Implications for the Profession and/or Patient Care:**

Sharing the narratives of nursing home staff with peers would support them in implementing interventions to foster trust. Regular Moral Case Deliberations can be used to address moral uncertainty. Collective dialogue among nursing home staff can be useful in establishing new social norms that prioritise family involvement. Conversation skills training can empower nursing home staff.

**Impact:**

Trust between nursing home staff and families can be improved by implementing the conducted interventions.

**Reporting Method:**

This report adheres to the standards for reporting qualitative research (COREQ).

**Patient or Public Contribution:**

No patient or public contribution.

**Summary**

**What Is Already Known**

- Families often stay involved in the care for their relative when he/she moves to a nursing home.
- Family involvement is a challenge for both family members and nursing home staff.
- Trust plays a central role in issues experienced in family involvement, emphasising the need for facilitating initiatives for building and maintaining a trusting relationship.

**What This Paper Adds**

- Self‐designed interventions of nursing home staff to foster a trusting relationship with families can contribute to family involvement and to quality of care.
- Initiating informal conversations, sharing ‘happy’ moments of residents, discussing mutual expectations and creating awareness of families' emotional burdens are experienced as positively contributing to a trusting relationship with family members.
- The barriers identified during the implementation of the interventions relate to moral uncertainty, conflicting social norms, and lack of courage to act.

**Implications for Practice/Policy**

- Narratives of the self‐designed interventions can be used for team reflection to assess the transferability to similar situations and improve quality of care.
- Moral case deliberation and collective dialogue is advised to minimise barriers.
- In addition, training nursing home staff in conversational skills with families can create self‐confidence and courage to engage in setting boundaries.

## 1 Introduction

After a relative moves to a nursing home, family members often continue to participate in their care (Bolt et al. 2019). For residents with dementia, family involvement is especially important, since they may have difficulty expressing their care needs and how they feel about the quality of the care they receive (Robison et al. 2007). Family members can offer valuable insights into care preferences and wishes of their relative, as well as how they perceive the care given, thereby helping nursing home staff to deliver personalised and relational care (McCormack et al. 2012). In addition, the provision of social–emotional support by family members to their relatives with dementia is essential to the resident's well‐being, because of the relationship they have established over time (Puurveen, Baumbusch, and Gandhi 2018). Among other benefits, it can help to make the resident feel at ease and comfortable (Hayward et al. 2022).

Nevertheless, family involvement often remains a challenge for both family members and nursing home staff. Studies highlight that from a family point of view, many issues arise when building good collaborative relationships with nursing home staff (Hoek et al. 2021; Hovenga et al. 2024). Examples include nursing home staff not taking the initiative to get to know one another (Hoek et al. 2021; Cottrell et al. 2018), inadequate discussion of mutual expectations (Jakobsen et al. 2019) and families not receiving sufficient information about their relative's wellbeing (Hoek et al. 2021; Omori et al. 2019). Moreover, the emotional and social difficulties that families go through can have a detrimental impact on their involvement in care. For instance, observing the mental decline of a relative (Wallerstedt et al. 2018), experiencing remorse about the placement decision (Lundh, Sandberg, and Nolan 2000) or juggling care and work‐related commitments (Barken and Lowndes 2018) can all interfere with families’ participation. Nursing home staff must cope with an increased workload due to staff shortages. They often feel that they do not have enough time to provide the desired level of care. This can result in moral stress and dissatisfaction among nursing home staff, which may cause tensions in their relationship with families (Preshaw et al. 2016). Furthermore, nursing home staff sometimes view families as too demanding, which can lead to reluctance to collaborate with them (Bauer et al. 2014).

## 2 Background

An interpretative synthesis of the literature from a care ethical perspective showed that trust played a central role in all issues of family involvement in nursing homes reported by families. The study concluded that initiatives to improve family involvement should therefore focus on strengthening trust in the relationship between nursing home staff and the family (Hovenga et al. 2022). Several other studies also indicate the importance of trust in the relationship between family and nursing home staff (Hoek et al. 2021; Jakobsen et al. 2019; Bauer et al. 2014; Majerovitz, Mollott, and Rudder 2009).

A review of the literature on the concept of trust between families and healthcare professionals identified several factors that can positively influence a trusting relationship, including a mutual intention to create trust, sufficient time to develop trust, reciprocity, absence of superficiality, mutual respect and honesty (Lynn‐McHale and Deatrick 2000). Also positively associated with trust is when nursing home staff in dementia special care units meet families' expectations of how their relative should be cared for and are attentive to residents' needs (Jakobsen et al. 2019; Lynn‐McHale and Deatrick 2000; Havreng‐Théry et al. 2021; Russell et al. 2021). In addition, informal contact between nursing home staff and family members appears to provide a good basis for a trusting relationship (Hoek et al. 2021). Nursing home staff who initiate an ongoing dialogue, focusing first on getting to know each other and then on aligning mutual expectations, can also be helpful in establishing and maintaining a trusting relationship with the family (Hovenga et al. 2022). These findings point to the need for facilitating initiatives for building and maintaining a trusting relationship between nursing home staff and family members. However, we found no research that explored the experiences of nursing home staff in integrating these elements into initiatives and implementing them in practice.

## 3 Methods

This study is part of a larger research project aimed at developing strategies to enhance family involvement in nursing homes. The initial phase of this larger research project, conducted in five special dementia care units in the Netherlands, explored the issues experienced by family members when participating in the care of their relatives (Hovenga et al. 2024). Findings revealed four central themes that caused tensions among family members: (1) limited familiarity with nursing home staff, (2) uncertainty about care expectations, (3) reluctance to contact or provide feedback to nursing home staff, and (4) difficulties coping with the relative's decline. In this follow‐up study, using a participatory action research (PAR) design, we asked nursing home staff how they would address these issues, resulting in various self‐designed interventions. This qualitative study reports on this iterative PAR process, and explores the experiences of nursing home staff in implementing their self‐designed interventions as well as how these interventions may foster trusting relationships with families in practice.

Results are presented in a co‐constructed narrative form, representing the experiences of nursing homes staff of the PAR process and lessons learned. By making these interventions visible and accessible, we aim to (1) enable readers to picture the experiences of nursing home staff and thereby develop new awareness of their own experiences, and (2) inspire them to initiate and implement own interventions to improve trusting relationships with family members in their own work environments.

### 3.1 Design

PAR was selected as the design of this study for multiple reasons. First, in PAR, participants and researchers work together to identify, analyse and resolve issues that are experienced in everyday practice (Kindon and Kesby 2007a; Kindon and Kesby 2007b). In this study, the issues of family members were presented and acknowledged by the nursing home staff as important to address in their own units. Second, PAR aims to contribute to a more democratic research process in which participants are involved as researchers, that is, they are invited to research the process themselves, instead of having research done on or for them. This approach actively engages participants to reflect and consider the successes and challenges of their self‐designed interventions during the implementation process (Grimwood 2015). Finally, PAR offers participants a chance to enhance skills, knowledge and competencies in addressing issues which can improve the sustainability of their self‐designed intervention (Kindon and Kesby 2007b).

### 3.2 Participants and Recruitment

Our study was conducted in five dementia special care units in nursing homes affiliated with the University Elderly Care Network of the University Medical Center Groningen in the north of the Netherlands. These five dementia special care units were selected because of their involvement in the initial phase of the larger research project, which identified issues experienced by family members caring for their relative. In line with PAR, each dementia special care unit was considered as a case study with its own culture, allowing participants to design and test their own intervention(s) to address the identified issues. In this way, PAR provides space to connect and respond to contextual factors and the participants involved, rather than following strict methodological frameworks of implementing prescribed interventions (Kindon and Kesby 2007b; Kindon 2005).

At the beginning of this study, managers from each of the five dementia special care units committed to participating in the project and identified and recruited one or two colleagues from their own dementia special care unit (hereafter referred to as local project leaders). Inclusion criteria were: (1) having regular contact with residents' families regarding daily care; (2) having experience guiding the implementation of improvement projects in practice; (3) being able and willing to share experiences on a regular basis with the researchers. Table 1 shows the characteristics of the local project leaders.

**TABLE 1:** Characteristics of the local project leaders.

Local project leaders (*n* = 8)	
Position in dementia special care unit	*n*
Coordinating nurse assistant	4
Wellbeing assistant	3
Nurse	1

These local project leaders recruited focus group participants within their units due to their thorough understanding of the unit's context and dynamics, enabling more targeted recruitment. In addition, their involvement in the recruitment process could increase their accountability and commitment, which could improve the success of implementing the self‐designed intervention. To achieve a variety of perspectives, family members as well as nursing home staff in different positions were selected. Family members were eligible if their relative with dementia lived in the nursing home. Nursing home staff were recruited based on their interest and involvement in the topic of this study. It is unknown how many potential participants indicated they did not want to participate in the study. Table 2 shows the characteristics of the focus group participants.

**TABLE 2:** Characteristics of the focus group participants.

Focus group participants (*n* = 78)	
Position in dementia special care unit	*n*
Nurse	16
Nurse assistant	14
Family member	8
Coordinating nurse assistant	7
Manager	6
Chaplain	6
Wellbeing assistant	6
Psychologist	5
Elderly care physician	3
Volunteer coordinator	3
Coordinating wellbeing assistant	2
Social work student	1
Physician assistant	1

### 3.3 Data Collection

The study consists of three phases. In the first phase, a heterogeneous focus group was conducted in each dementia special care unit (*n* = 5) to (1) identify interventions to address the previously identified issues with the aim to build and maintain a trusting relationship with family, (2) select one of these self‐designed interventions to implement in practice over a period of 4–5 months, and (3) outline the key elements of these intervention(s), including objectives, actions, responsibilities, risk management and evaluation criteria. The focus groups were conducted face‐to‐face in a meeting room at the dementia special care units, consisted of four to eight participants and lasted 2.5 h.

In the second phase, together with the researchers, the local project leaders further redefined their chosen intervention(s). In the third phase, the local project leaders started the process of coordinating and implementing their intervention(s) in their own dementia special care unit. This implementation process was jointly evaluated by the researchers (NH, FV, IL) and the participants consisting of the following three methods.

First, the local project leaders were interviewed every 2–3 weeks, individually (*n* = 13) or in pairs (*n* = 15), by one of the researchers (NH, FV, and IL) via online video conferencing. The mean duration of the individual interviews was 30 min (range: 10–95 min). Prior to the interviews, the local project leaders were asked to record their experiences of conducting the intervention throughout the project. These notes were used as reminders during the interviews. An open‐ended, narrative interview structure was used in which narratives were produced and interpreted interactively between researchers and participants (Scheffelaar, Janssen, and Luijkx 2021). In accordance with the method of open narrative interviewing, the researchers started the interviews by presenting an open‐ended question to encourage the local project leaders to share their experiences in their own words. The question was: ‘What were your experiences with implementing your intervention over the past few weeks?’ During the first part of the interview, the interviewer's interruptions were kept to a minimum. In the second part of the interview, the interviewers asked questions that were aimed at elucidating the narrative, such as asking for specific examples to clarify the story (Scheffelaar, Janssen, and Luijkx 2021). In addition, the interviewers assisted the local project leaders in analysing their reflections, with a focus on identifying barriers and facilitators. They were also encouraged to find solutions to overcome the barriers they experienced. The narrative interview method aligns with the PAR design of the study, in which interviewers do not adopt the expert role, but instead facilitate collaborative learning (Kindon 2005). The interview guide is included in Table 3.

**TABLE 3:** Interview guide for individual or paired interviews with local project leaders.

What were your experiences with implementing your intervention over the past few weeks?
What were your observations? What stood out? What challenges have you faced? How did you deal with them? What went smoothly? What factors made it easier? What are you satisfied with? And why? What do you think family members and colleagues have noticed about your actions? How do these results relate to the goals you set before?
How would you like to proceed with the implementation process in the next few weeks?
Do you see areas for improvement? If so, which ones? What difficulties do you foresee? How do you plan to deal with them? What will it take to make things easier?

Second, online focus groups were conducted halfway through (*n* = 3) and/or at the end (*n* = 5) of the PAR period, consisted of two to seven participants and lasted an average of 50 min (range 42–64 min). In the focus groups held during the midpoint of the PAR period, the participants reflected on their experiences with the previously taken actions. Their goal was to collaboratively determine the implications of these actions for next steps to be taken. Topics addressed were (Bolt et al. 2019) barriers and facilitators in the implementation process, (Robison et al. 2007) identified benefits of the intervention, and (McCormack et al. 2012) suggestions for follow‐up actions. The main topics of the online focus groups held at the end of the research period were (Bolt et al. 2019) understanding identified benefits and (Robison et al. 2007) identifying overall lessons learned, including recommendations for nursing home staff from other units or organisations who may also want to implement the intervention.

Third, the researchers (NH and/or FV) were invited by local project leaders from three dementia special care units to observe one or more online team meetings attended by nurse assistants during which the intervention was discussed (*n* = 5). The researchers (NH and/or FV) did not actively participate, but took detailed field notes about what actually happened during these team meetings. The researchers then reflected on their observations with each other and with the local project leaders in a following interview round.

All focus groups (*n* = 15) were led by a researcher who was assisted by another researcher (NH, FV, IL, EL). Detailed reports of the individual interviews and focus groups were written based on the audio recordings and were pseudonymised. The data collection period began in August 2021 and ended in April 2022.

### 3.4 Data Analysis

A holistic narrative analysis (Scheffelaar, Janssen, and Luijkx 2021; Elliott 2005) was conducted, consisting of two steps. In the first step, the researchers (NH, FV, and IL) read and re‐read all data collected from each of the six conducted interventions several times, separately and in detail. Together with the participants they then selected and organised key elements of the stories participants told about themselves and their experiences. They used the main topics of the (group) interviews—(i.e., description and subgoals of the intervention, barriers and facilitators, identified benefits, and recommendations) as sensitising concepts. In doing so, participants’ stories were kept intact, resulting in first‐order narratives (Elliott 2005).

In the second step, the researchers (NH, FV, IL, and EL) collaborated with the local project leaders to construct second‐order narratives by retell or ‘restory’ participants first‐order narratives into a meaningful whole (Elliott 2005). This process of analysis resulted in six second‐order narratives, as one dementia special care unit implemented two different interventions simultaneously as they thought it would be feasible. To avoid repetition, the research team (NH, FV, IL, EL, SZ, and CL) together with the local project leaders agreed to merge four of the six narratives into two as they were describing experiences related to implementing similar interventions. The four co‐constructed second‐order narratives were seen as the product and end point of analysis in the research process (Etherington 2002).

### 3.5 Rigour

The researchers used various strategies to improve the credibility of this study. First, triangulation was achieved by utilising various methods of data collection, including individual interviews, focus groups and observations. In addition, including different perspectives resulted in a variety of data sources. Second, the researchers (NH, FV, IL, ST, EL) analysed the data independently and discussed the preliminary findings with each other on a regular basis, also referred to as peer debriefing. Third, in order to prevent bias, the researchers (NH, FV, IL, ST, EL) and participants reflected on the ways in which the context and their interactions shaped the collection of data, including the role of prior assumptions and experiences (Mays and Pope 2000). Fourth, all individual interviews and focus group reports, as well as the second‐order narratives, were returned to the participants for a member check. This allowed participants to evaluate the researchers' accuracy and credibility (Mays and Pope 2000). The content was approved by all participants.

### 3.6 Ethical Considerations

The study and its protocols were ethically approved by the Central Ethics Review board of the University Medical Center Groningen (CTc UMCG, 201900374).

To obtain informed consent, potential participants received a letter explaining the purpose of the study. After local project leaders indicated they were interested in participating, they met with one of the researchers (NH, FV, IL). A more detailed explanation of the study was given, and research‐related questions were answered. In addition, the researchers introduced themselves, sharing their background and motivation for engaging in the research topic. Written informed consent was obtained from all participants prior to the (focus group) interviews.

During the focus groups, the researchers paid attention especially to the development of an open and trusting atmosphere in which all participants could let their voices be heard. For example, by emphasising that the focus group reports would be pseudonymised and the audiotaped interviews would be confidentially managed throughout the study. Pseudonyms were used in the second‐order narratives (end‐product of the analysis process) to ensure that the identities of local project leaders and other participants remained confidential.

## 4 Results

Below, we present the four second‐order narratives based on participants' experiences of conducting the following interventions: (1) initiating informal conversations, (2) sharing ‘happy’ moments of residents, (3) discussing mutual expectations, and (4) creating awareness of families' emotional burdens. Participants identified and selected these interventions based on their potential to positively contribute to foster a trusting relationship with family members and their feasibility of implementation within four to five months in their dementia special care unit. The narratives consist of the following components: description and subgoals of the intervention, barriers and facilitators, benefits, and recommendations. The narratives are written in the third person from the perspective of the local project leaders. To increase the sense of familiarity, we have included quotes in these narratives.

### 4.1 Initiating Informal Conversations

Rose (nurse) and Alice (wellbeing assistant) are working in two different dementia special care units. They started to initiate informal conversations with family members more often and encouraged their immediate colleagues on the team to do the same.

Rose and Alice state that an informal conversation with family can be about anything and has no set agenda. In particular, it is a way to show personal interest in family members. Rose points out that she sometimes shares a little bit about her own personal life too, for example about her mother‐in‐law who also lives in a dementia special care unit.

Rose: *Through the sharing of personal experiences during informal family contact, the other person begins to see you as a human being rather than a caregiver*.

At the same time, Rose wants to be careful about what kind of private information she shares with family because it can put her in a vulnerable position. Rose notices that when she is too open, family may think that she will meet all their needs and expectations, which is often neither desirable nor possible.

Rose: *A family member once asked me if they could call me at home at night. At that moment, I said I didn't want them to. It would cross my personal boundaries*.

Due to staffing shortages on the ward, Rose, Alice and some of their colleagues encounter the dilemma of initiating informal contact with family members while at the same time other residents need their attention. Alice notices that some colleagues choose not to prioritise chatting with family members, because they view it as an extra task, not as part of their job.

Alice: *If you leave the dishes to talk to family, that is your job, but it is not perceived that way by everyone*.

Alice and Rose sometimes struggle to convince their colleagues to have a chat with family members more often. Many colleagues say they already have regular conversations with family members and therefore see no need to change their behaviour. But Alice and Rose have a different experience. Especially when it comes to families who are critical, angry, or sad. Then they observe a certain reluctance among colleagues to approach family for a chat.

Rose and Alice consider initiating informal contact with family members on a regular basis very beneficial. For example, they feel family members are more comfortable approaching them when they are struggling with something, which can help to avoid tension in the relationship. Rose adds that she recently asked a resident's son how he was doing. They then had a very nice conversation, during which she learned more about his mother's care needs. Rose and Alice both agree that regular, informal and personal contact with family members generally contributes to family members’ feelings of recognition. They are better informed about what their relative's daily life in the nursing home is like and feel more welcome and involved in their relative's care in the nursing home.

Alice: *Ultimately, it is important that family comes to visit us with a good feeling and that there is mutual trust. Therefore, we must make an extra effort to get to know each other*.

### 4.2 Sharing ‘Happy’ Moments of Residents

Nora and Ivy, both wellbeing assistants, are employed in two different dementia special care units. Together with several other colleagues, they started to share (more) photos and/or descriptions of residents' happy moments with family members spontaneously. For example, Ivy reports to the family that their relative had been humming at the table after she had spent more time with him than usual. Nora and Ivy experience that family members like to recognise their relatives in the shared ‘happy moments’.

Nora: *Then they say ‘Oh how funny, that's typical of my mother, fun to read!’*

It is also nice to share special moments of residents' togetherness, although Nora sometimes wonders if she is doing the right thing.

Nora: *I hesitated to post a photograph of two residents walking hand in hand because one of the residents still had a partner. You could hurt that partner unnecessarily by sharing such a photo*.

Ivy also notes that what you report about resident's behaviour may be painful to family members, so you should always be aware of the impact of a report on family members. Nora states that nurse assistants often think that sharing the resident's ‘little daily life things’ is not that valuable for family members. Her experience is different:

Nora: *My experience is that these ‘little things’ are also very nice for family members to know. That is why it is important to ask yourself what you would like to know if your relative lived in a nursing home*.

It is a challenge for some colleagues to find the right words to describe a resident's happy moment. To them Nora says that just sharing a photo is also fine.

Ivy notices that nurse assistants tend to report things that are not going well, such as a resident being aggressive to a fellow resident. There is nothing wrong with that, but it is important to write down how you handled it. Family members as well as colleagues will know that you have thought about how to solve the problem in a good way. Ivy points out that it is also a positive thing to write down tips that you get from family members in the file so that they can read them again later. One tip might be how to reassure the person's relative when he/she is upset. Ivy's experience with family members is that it makes them feel recognised and taken seriously.

Nora and Ivy notice that nurse assistants focus mainly on care needs, such as washing, dressing, wound care and medication administration, and then report on them.

Ivy: *Most nurse assistants have little experience reporting on residents' happy moments. They focus more on the care needs of the residents and less on the well‐being aspect. They also say they haven't got time for it, but I think it should be part of their job*.

Ivy and Nora feel good when they see that family members are happy with a message or photo they have shared. Nora points out that good care is not just about the resident, but also about the family. Nora and Ivy note that in their experience sharing moments of happiness is often an easy way to start an informal conversation with family. This approach allows them to get to know each other better. As a result, family members have more confidence in them.

### 4.3 Discussing Mutual Expectations

Kate and one of her colleagues, both coordinating nurse assistants, began discussing mutual expectations with family members more often in their dementia special care unit.

Kate shares her experience of discussing mutual expectations with a family member of a woman who has hit nurse assistants several times when they tried to get her out of bed. Still, the son wanted his mother to get out of bed more often to participate in daily activities. But the resident indicated she did not want to.

Kate: *We were faced with the dilemma of getting her out of bed against her will or letting her stay in bed more often against her son's wishes*.

Unlike before, Kate takes the initiative to discuss this dilemma with the son. During one of those conversations, the son explains how they could best approach his mother to get her out of bed. Kate emphasises the need for herself and her colleagues to avoid getting physically injured while caring for his mother and to be respectful of the mother's wish to remain in bed.

Kate: *As a result of these conversations, we were able to be more responsive to Mrs X. and get her out of bed more often, and it was clear to the son what our boundaries were and what was feasible for us in terms of his mother's care*.

It is sometimes difficult for Kate to communicate to family members what she expects from them regarding caring for their relative. She does not want to put an extra burden on family members, who are often already exhausted from providing care to their relative at home. In addition, she assumes they often have other responsibilities and therefore do not have the time or opportunity to be more involved in their relative's care in their unit.

Kate notes that it is easier to discuss more difficult topics, such as mutual expectations, when you know each other better. Kate found that providing clarity about what you can expect from each other is the most important benefit of discussing mutual expectations with family members.

Kate: *By being more explicit about expectations and boundaries, family members better understand that sometimes we can't do more than we can to care for their relative. Conversely, we have more sympathy for the family that sometimes their involvement in their relative's care is limited because they are sometimes overwhelmed*.

Kate said that a daughter of a resident indicated that by discussing mutual expectations with her more often, the lines of communication became shorter, giving her a better understanding of how we provide care.

Daughter: *It reassures me when I hear that my father is doing well because you always wonder*.

### 4.4 Creating Awareness of Families' Emotional Burdens

Families often experience feelings of grief and other emotional challenges when their relative is admitted to a dementia special care unit, says Emma (coordinating nurse assistant). For example, it can be emotionally difficult for family members to no longer be recognised by their relative.

Emma: *Overall, a family must deal with loss every time. Or as one social worker put it*:

*‘The Band‐Aid is taken off slowly.’*

Emma is not surprised that family members sometimes overreact to things that may not be very important in the eyes of nursing home staff. For example, whether the frames of family photos are in the right place or how the laundry is folded and stored in the closet. Often there is an emotion or a cry for help behind the family's reaction. Emma is convinced that the relationship with family will improve if her colleagues become more aware of experiences of loss and grief, show understanding and try to find out exactly what is at stake for families.

Emma wants to encourage the nurse assistants on her team and the other teams at her site to be more aware of the family's experience of loss and grief. To that end, Emma invites the social worker to share information and initiate a discussion on this topic during one of the regular team meetings. During these team meetings, the social worker gives a presentation on what grief is, how to recognise it, and how to deal with it. Most nurse assistants say they find the information relatable.

Nurse assistant: *You know all these things, but it is good to clarify the issue and to think about it again*.

Emma reports that the conversation during the team meetings started when the nurse assistants were invited by the social worker to share their own experiences with so‐called ‘difficult’ families. For example, a nurse assistant told of a resident's spouse who regularly becomes angry when things do not go the way she wants. The nurse assistant said she understood this behaviour. But she also found it difficult to deal with it. Together they explored what might be going on with this relative and what she might need.

Later, some of the nurse assistants tell Emma that they start to think more about how it would feel for family members to have their relative living in their dementia special care unit, and that they are now trying to find out more often what is important to them in their relative's care.

In this context, one of Emma's fellow coordinating nurse assistants emphasises the importance of sharing residents' happy moments with families. According to her, sharing a nice anecdote can build a closer bond with relatives, which makes it easier for both staff and relatives to also discuss emotional difficulties.

Coordinating Nurse Assistants: *The bit of fun makes the bit of grieving more bearable and easier for family*.

Emma has encouraged her colleagues to ask family members how they are doing more often. In this way, Emma explains, they may be able to create an opening for conversation, find out the reason for certain emotional behaviours, and learn how to deal with family members they sometimes perceive as ‘difficult’.

## 5 Discussion

During the implementation of all four interventions, local project leaders strived for more reciprocity in their relationship with family members by bringing more intimacy into their interactions (intervention 1), sharing positive impact of their caregiving activities on their own initiative (intervention 2), setting boundaries for caregiving responsibilities (intervention 3), and creating an empathetic, compassionate relationship with family members (intervention 4). Previous literature indicates that reciprocity in the relationship between the health care worker and the family is an important prerequisite for trust, because it can help to make the power inequality between the one who trusts and the trustee visible and more balanced (Lynn‐McHale and Deatrick 2000; Baier 1986; Adam and Donelson 2022). In addition, the findings show that the interventions are interrelated in the sense that they can be mutually supportive in fostering a trusting relationship with family. For example, Ivy and Nora indicate that it is easier to initiate an informal conversation with family (intervention 1) if you can refer to a shared ‘happy’ moment of their relative (intervention 2). The overarching outcome of the interventions was that participants experienced an improved level of connectedness with family members. This supports the findings from previous research that it is worthwhile to include the facilitating factor of ‘knowing each other’ or ‘connecting with each other’ in interventions to be implemented in daily practice (Hovenga et al. 2022; Lynn‐McHale and Deatrick 2000). In the next paragraph, we reflect on the findings and put them in dialogue with the existing empirical and theoretical literature. We begin by examining the experiences with each intervention individually and then focus on the barriers.

Rose and Alice tried to build a trusting relationship with family members by initiating more informal contact and sharing more personal information about their private lives, such as having a relative living in a nursing home as well (intervention 1). Above all, Rose and Alice emphasised their desire to feel connected to family and to be recognised as a fellow human being and not just as a professional who focuses primarily on care issues. In one study, this approach was defined as a ‘friendship’ relationship between nursing home staff and the family (Gladstone and Wexler 2002). This expression of commitment by professionals to relate to a resident as fellow human being brings the (vulnerable) other to the foreground as someone who is worth the effort and with whom one feels connected (van Heijst 2011). Rose and Alices’ strategy seems to stem from their belief that family members find it much easier to trust them if they express their willingness to enter into a ‘friendship’ relationship with them. Previous research supports that familiarity positively affects families’ level of trust in their relationship with nursing home staff (Bauer et al. 2014; Majerovitz, Mollott, and Rudder 2009; Russell et al. 2021). One possible explanation for this is that it provides family members with a deeper insight into the intentions and motives of nursing home staff. This is important for family members in order to assess the extent to which nursing home staff will act in the best interests of their relatives, or in other words, can be trusted (Baier 1986).

Nora and Ivy's focus was on share residents' ‘happy’ moments with family members (intervention 2). Family members expressed their desire to recognise their relative in these shared ‘happy’ moments. Maintaining the resident's identity seems to reflect the family's need to have their relative's preferences respected by nursing home staff, which is positively related to trust (Hoek et al. 2021; Hovenga et al. 2022). During the research project, Nora and Ivy also chose to report in more detail on their responses to situations where the resident was dissatisfied, for example, how they comforted a resident who was agitated. This strategy seems to be a way for Nora and Ivy to present a realistic picture and show family members that their relative is also being well taken care of in case of more difficult situations. Previous literature suggests that family members' trust in nursing home staff increases when the latter provide unsolicited (positive) information about their relative's well‐being (Omori et al. 2019). Additionally, this approach seems to be another expression of the nursing home staff's dedication to family members and their relatives. Nursing home staff showing their competence and goodwill in this way helps families to trust them (Baier 1986).

Kate described her experiences discussing mutual expectations with family members (intervention 3). Among other things, she says, this is an opportunity to find out what is important to family members in approaching and caring for their relative in the dementia special care unit. This corresponds to various studies indicating that when nursing home staff listen to and respect family members' expectations, a constructive and lasting relationship of trust is fostered (Hovenga et al. 2024; Bauer et al. 2014; Havreng‐Théry et al. 2021). According to Kate, this strategy is also a good opportunity to share with each other limits of what is possible and to set expectations that are more in line with reality. This is supported by research. Discussing boundaries can prevent family and nursing home staff from having expectations of each other that are not met, which in turn can lead to feelings of powerlessness and mistrust (Jakobsen et al. 2019).

Emma strived to sensitise her colleagues to the emotional burdens that family members can face after their relative is admitted to a dementia special care unit. According to Emma, this strategy stimulates the ability of her colleagues to empathise with the family's situation. Developing compassionate interactions based on empathy, encouragement and emotional support to help family members cope with ongoing issues related to nursing home placement is positively related to trust. Family members may feel more supported rather than left alone with the complexity of grief and loss (Puurveen, Baumbusch, and Gandhi 2018).

Barriers identified by local project leaders during the process of implementing their interventions can be related to (1) moral uncertainty, (2) conflicting social norms, and (3) lack of courage to act. Moral uncertainty can be defined as being uncertain about what you ought to do when you do not know what to do (Sepielli 2014). For example, Rose and Alice (intervention 1) experience moral uncertainty as they try to make informal contact with family while facing demands from other residents. Kate (intervention 2) said that she sometimes has doubts about sharing certain photos of residents' happy moments with family members because they may hurt their feelings. Local project leaders' doubts about the definition of good and what is good for whom may have hindered the implementation of all four interventions to some extent. Nursing home staff face moral dilemmas about quality of life and care every day (Bolmsjo, Edberg, and Sandman 2006). This has been identified as one of the barriers to implementing interventions in long‐term care (McArthur et al. 2021).

Social norms can be experienced as a barrier when they conflict with desired behaviour related to an intervention (McArthur et al. 2021; Bicchieri 2016). A social norm exists when people's behaviour is influenced by what relevant others are doing and think should be done (Bicchieri 2016). Rose and Kate (intervention 1) say that most of their colleagues do not always prioritise family contact because they do not consider it as part of their job. This behaviour pattern, or social norm, is also illustrated during the implementation of the second intervention, where Nora and Ivy note that most of their colleagues, when faced with time constraints, prioritise completing care tasks rather than sharing ‘residents’ happy moments' with family members. The social norm of prioritising tasks is in line with previous research indicating that nurse aides consider care mainly in terms of physical activities, with comparatively limited attention given to social and emotional care (Balkin et al. 2023).

Participants sometimes experience setting boundaries on what family members can expect from them in caring for their relative as a challenge. For example, Rose (intervention 1) was hesitant about how far she could go in sharing private information in order to avoid unrealistic expectations on the part of family members, and about having to define her boundaries. Kate (intervention 3) also noted that setting boundaries sometimes hindered her discussing mutual expectations with family members. In addition, Alice and Rose (intervention 1), like Emma (intervention 4), note that their colleagues sometimes avoid initiating a conversation with family members who are angry, sad, and/or critical. Participants struggled with overcoming fear to act in a way they believe is right, in other words may lack courage to act. In literature, courage is recognised as an important component of nursing practice and can be defined as the ability to overcome challenges such as fear and dislike, and to face moral uncertainty about one's capabilities (Spence and Smythe 2007).

## 6 Strengths and Limitations

To our knowledge, this is the first study to narrate the participants' experiences of conducting co‐designed interventions to foster trusting relationships with family members in dementia special care units.

A strength of this study is that the four narratives may provide readers with alternative perspectives on how to foster trusting relationships with family, potentially inspiring them to initiate similar interventions and tailer them to their own work environments. At the same time, the extent to which the interventions can be transferred beyond the setting of the dementia special care unit setting may be limited. To address this concern, we have provided a detailed account of the experience of implementing the interventions, enabling readers to assess the transferability of these interventions to similar situations (Mays and Pope 2000).

Another limitation of the study is that the local project leaders were responsible for recruiting family members for the focus groups. As a result, there is a possibility that they selected primarily family members with whom they had a good relationship. This may have resulted in a limited diversity of experiences with the interventions studied.

## 7 Recommendations for Practice and Future Research

It is suggested to nursing home staff of dementia special care units (or other comparable working environments) to consider implementing intervention(s) similar to those described in the four narratives in order to contribute to a trusting relationship with family members. To promote and support nursing home staff in this regard, we compiled the four narratives into a co‐designed package, including illustrations. We subsequently disseminated this package among nursing homes and nursing educational institutions in the Netherlands with the suggestion to reflect on them in a group setting. The collected narratives can be used for team reflection to improve quality of care (Scheffelaar, Janssen, and Luijkx 2021).

To minimise experienced barriers to implementing one or more interventions, we recommend the following overarching actions, based on participants' suggestions from the focus groups held at the end of the research period and reflections on the barriers discussed. First, to overcome the barriers related to moral uncertainty, we recommend organising Moral Case Deliberation (MCD) on a regular basis, where nursing home staff are given the opportunity to reflect on their moral issues related to fostering a trusting relationship with family members. MCD has proven its relevance in practice. For example, it strengthens the moral competence of health care professionals, enabling them to make difficult and painful decisions (Spronk, Widdershoven, and Alma 2020). Good implementation of MCD is achieved when, among other things, management ensures that MCD activities are budgeted for and led by a trained MCD facilitator (Hartman et al. 2022).

Second, to overcome barriers related to dominant social norms, in this case the social norm of nursing home staff to prioritise physical care tasks over connecting with family members, shared perspectives are needed. To change a social norm requires that the reasons for change must be collectively shared (Bicchieri 2016; Walker 2007). Collective dialogue is considered a powerful tool for this, because it may uncover the rich web of beliefs, values, scripts and mutual expectations that constitute the social norm and disclose conflicts or inconsistencies that could be addressed together (Bicchieri 2016; Walker 2007). So, a dialogue among nursing home staff could reveal why the task‐oriented approach may contradict certain core institutional and personal beliefs. In addition, for this social norm to change, there must be a mutually shared consensus to abandon the old norm, to agree on a new practice, and to promise to follow it (Bicchieri 2016).

Third, courage to act should be fostered among nursing home staff to give them the strength to overcome barriers related to fostering a trusting relationship with family members. It may be beneficial to train nursing home staff in conversational skills to (1) set boundaries in their relationship with family members and (2) engage in conversations with families that they perceive as ‘difficult’. Such training can boost their self‐confidence and decrease their fear of taking action, ultimately reducing the need for courage. Courage in nursing is a complex phenomenon that manifests itself in different ways in practice (Lindh et al. 2010). This may be the reason why, as far as we know, there is little knowledge about how to support courage in nursing practice. Therefore, we suggest focusing future research on this topic.

## 8 Conclusion

Initiating informal conversations, sharing ‘happy’ moments of residents, discussing mutual expectations, and creating awareness of families' emotional burdens are experienced as positively contributing to a trusting relationship with family members. The barriers identified during the study related to moral uncertainty, conflicting social norms and lack of courage to act. Sharing nursing home staff's experiences with peers and interventions to overcome the ethically loaded barriers are recommended to improve family involvement in practice.

## Author Contributions

Made substantial contributions to conception and design, or acquisition of data, or analysis and interpretation of data: Nina Hovenga, Elleke Landeweer, Ivonne Lesman‐Leegte, Sacha Van Twillert, Floor Vinckers, Sytse Zuidema and Carlo Leget. Involved in drafting the manuscript or revising it critically for important intellectual content: Nina Hovenga, Elleke Landeweer, Ivonne Lesman‐Leegte, Sacha Van Twillert, Floor Vinckers, Sytse Zuidema and Carlo Leget. Given final approval of the version to be published. Each author should have participated sufficiently in the work to take public responsibility for appropriate portions of the content: Nina Hovenga, Elleke Landeweer, Ivonne Lesman‐Leegte, Sacha Van Twillert, Floor Vinckers, Sytse Zuidema and Carlo Leget. Agreed to be accountable for all aspects of the work in ensuring that questions related to the accuracy or integrity of any part of the work are appropriately investigated and resolved: Nina Hovenga, Elleke Landeweer, Ivonne Lesman‐Leegte, Sacha Van Twillert, Floor Vinckers, Sytse Zuidema and Carlo Leget.

## Conflicts of Interest

The authors declare no conflicts of interest.

### Peer Review

The peer review history for this article is available at https://www.webofscience.com/api/gateway/wos/peer‐review/10.1111/jan.16432.

## Data Availability

The data that support the findings of this study are available on request from the corresponding author. The data are not publicly available due to privacy or ethical restrictions.
